# Microstructure Evolution and Properties Tailoring of Rheo-Extruded Al-Sc-Zr-Fe Conductor via Thermo-Mechanical Treatment

**DOI:** 10.3390/ma13040845

**Published:** 2020-02-13

**Authors:** Di Tie, Yu Wang, Xiang Wang, Renguo Guan, Lufei Yan, Jin Zhang, Zhihui Cai, Yang Zhao, Fei Gao, Haifeng Liu

**Affiliations:** 1 Key Laboratory of Lightweight Structural Materials Liaoning Province, School of Materials Science and Engineering, Northeastern University, Shenyang 110819, China; tie-di@hotmail.com (D.T.); caizh@mail.neu.edu.cn (Z.C.); zhaoy@mail.neu.edu.cn (Y.Z.);; 2 School of Materials Science and Engineering, Northwestern Polytechnical University, Xi’an 710072, China; jinzhang@nwpu.edu.cn; 3 The CITIC Dicastal Institute, Qinhuangdao 066011, China; liuhaifeng@dicastal.com

**Keywords:** Al-Sc-Zr-Fe conductor, rheo-extrusion, thermo-mechanical treatment, mechanical properties, conductivity

## Abstract

Low-cost heat-resistant Al-Sc-Zr-Fe conductor wires were successfully manufactured by continuous rheo-extrusion process, and the mechanical and conductive properties of the materials were analyzed and compared after three different thermo-mechanical treatment methods. The coarse plate-shape Al_3_Fe phase transformed to small sized rod-like phase after solid solution treatment at 630 °C for 21 h. Direct aging treatment at 300 °C for 24 h led to the refinement and spheroidization of Al_3_Fe phase with a diameter of 200 nm. After the subsequent aging treatment at 300 °C for 24 h, the tensile strength and conductivity of the alloy wire significantly increased due to the homogeneous precipitation of the coherent spherical Al_3_(Sc, Zr) phase with an average size of 15 nm. The tensile strength, elongation, and conductivity of the alloy conductor wire after optimized thermo-mechanical treatment reached 165.7 MPa, 7.3%, and 60.26% International Annealed Copper Standard (IACS), respectively. The thermal resistance of the present alloy wire was superior to that of standard AT1 type alloy conductor according to IEC international standard.

## 1. Introduction

Aluminum alloys have shown great potential for use as an electrical material owing to its light weight, good mechanical properties, suitable corrosion resistance, and excellent electrical conductivity [1,2]. Since the heat resistant effect of Zr addition into Al matrix was found, the development of the Al-Zr alloy conductor has drawn the attention of conductive material researchers [3]. Sc is also an effective solution strengthening element for Al alloys as reported by Davydov et al. [4]. Ocenasek and Slamova denoted that the addition of Sc and Zr in Al alloys could improve the recrystallization temperature of the alloys, thus improved the heat resistance and mechanical properties of the alloy at elevated temperatures [5]. The significant improvement of the mechanical properties of Al alloys containing Sc and Zr is due to the formation of nano-scale, coherent, and L1_2_ structure Al_3_Sc or Al_3_(Sc, Zr) precipitates [6]. Detailed investigations on dilute Al-Sc-Zr alloys showed that the Al_3_(Sc, Zr) phase consists of a Sc enriched core surrounded by a Zr enriched shell, as presented Tolley et al. [7]. The high thermal stability of Al_3_(Sc, Zr) phase makes the Al-Sc-Zr alloy materials have a huge potential of application at higher temperatures [8].

At present, a series of high-performance Al-Sc-Zr heat-resistant alloy wires have been developed [9]. However, high price of Sc and Zr elements results in the high cost of the Al-Sc-Zr alloy wire, which limits its application. The Al-Fe intermetallic compounds was found to have good heat resistance, abrasion, and corrosion resistance [10]. On the other hand, heat treatment and plastic deformation processing were proved effective ways to improve mechanical and conductive performance of Al alloy conductor [11,12]. Shen et al. reported significant improvement of mechanical properties were achieved after aging treatment to Al-Sc-Zr alloy at 600 °C for 36 h [13]. However, for Al-Fe alloys, heat treatment at that high temperature could arise coarsening phenomenon of Al-Fe phases [14]. In addition, the evolution mechanism of Al_3_Fe and Al_3_(Sc, Zr) phases during heat treatment and mechanical deformation is still unclear. To solve these problems, the expensive Sc and Zr in the Al-Sc-Zr alloy conductor was partially replaced by Fe in our study, and the preparation route of low-cost heat-resistant alloy wire was explored. The deformation behaviors of Al_3_Fe and Al_3_(Sc, Zr) phases during thermo-mechanical treatment as well as their effects on mechanical and conductive properties of Al-Sc-Zr-Fe conductor were revealed.

## 2. Materials and Methods

Al-0.05Sc-0.1Zr-0.2Fe (mass%) alloy was prepared by appropriate proportion of pure aluminum (99.99 mass%, Shandong Pingyin Xinsheng Aluminum Co., Ltd., Shandong, Jinan, China), pure iron (99.99 mass%, Taiyuan Steel, Shanxi, Taiyuan, China), Al-2Sc (wt%) master alloy, and Al-4.6Zr (wt%) master alloy (Zouping Huixin Metallurgical Materials Co., Ltd., Shandong, China). Materials were heated with specific ratios at 740 °C for 1200 s in a medium-frequency induction furnace (KGPS50-2, Yuanlu, Shanghai, China), under a protective atmosphere of argon gas. Chemical composition of the alloy was confirmed by inductively coupled plasma optical emission spectrometry (ICP-OES, Agilent 5100, Santa Clara, CA, USA).

**Figure 1:**
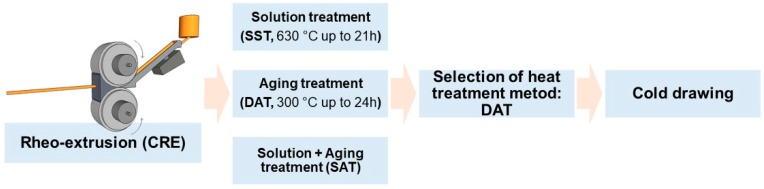
Schematic diagram of the processing and heat treatment and mechanical deformation routes.

The melt was purged at 710 °C and poured into a continuous rheo-extrusion (CRE) equipment developed by Guan et al. at 730 °C to prepare Al-Sc-Zr-Fe alloy wire with the diameter of 10 mm [15]. Part of the alloy wires were subjected to a solid solution treatment (SST) at 630 °C for 21 h, and then followed by an artificial aging treatment (solution treatment followed by aging treatment (SAT)) at 300 °C for 24 h. The other alloy wires were subjected to a direct aging treatment (DAT) at 300 °C for 24 h. Aging treatment time was longer than that of solution treatment to obtain better precipitation strengthening effect. The cooling rate after solution treatment was ca. 120 K/s, while the cooling rate after aging treatment was ca. 105 K/s. All the heated samples were quenched in the water at 20 °C, and the whole process route is depicted in Figure 1. The parameters of the heat treatments are shown in Table 1. The DAT treated alloy wires were cold drawn from 10 mm to 4.5 mm through 11 passes, using a 3 m chain drawing machine with carbide die. The drawing speed was 0.10 m·s^−1^. Diameters of the wires after different drawing passes are shown in Table 2.

Specimens were cut from the cross section of the alloy wires for microstructural characterization. Measured surfaces were ground with water and ethanol (>99.5%, Fuyu, Tianjin, China) down to a grinding size of grade 3200, polished with a lubricant containing 3 µm diamond particles (>99.5%, Jingxian, Shenzhen, China), and followed by a lubricant containing 1 µm diamond particles under addition of dish liquid (Jingxian, Shenzhen, China). In the last step of polishing, ethanol was used as lubricant instead of water. The specimen were then degreased and rinsed with pure ethanol. Then they were etched by using 0.5% HF (vol.%) aqueous solution. The optical microstructure was characterized with an optical microscope (DSX500, Olympus, Tokyo, Japan). The scanning electron microscopy (SEM) images were taken by a field emission scanning electron microscope (FE-SEM, SSX-550, Shimadzu, Kyoto, Japan). For transmission electron microscopy (TEM, Tecnai G2, FEI Company, Eindhoven, The Netherlands) observation, specimens were mechanically ground to the thickness of ca. 120 μm and then twin-jet-electropolished in a solution of 30% HNO_3_ and 70% CH_3_OH at −25 °C with the current of 60–80 mA. A CMT5105 electronic tensile testing machine (MTS Co., Fort Lauderdale, FL, USA) was used to measure the mechanical properties at room temperature, the tensile rate was set as 2 mm·min^−1^. Tensile tests were also carried out at 230 °C and 400 °C to test the thermal-resistant property using the tensile testing machine (MTS Co., Eden Prairie, MN, USA). The resistance of each sample was tested by a PC36C digital DC resistance tester (Hanyi, Shanghai, China) for three different lengths, and the conductivity of the wire was calculated based on Equations (1) and (2).

ρ = R·S/L(1)

K = 0.017241/ρ × 100%(2)

In which, *ρ*’s unit is Ω·mm^2^·m^−1^; *R* is resistance (Ω); *S* is the cross section (mm^2^); *L* is the length (m); and *K* is electrical conductivity as determined by the International Annealed Copper Standard (IACS). All the tensile and conductivity tests were carried out in five-duplicates, and the data were expressed as mean ± standard deviation (SD).

## 3. Results

**Figure 2:**
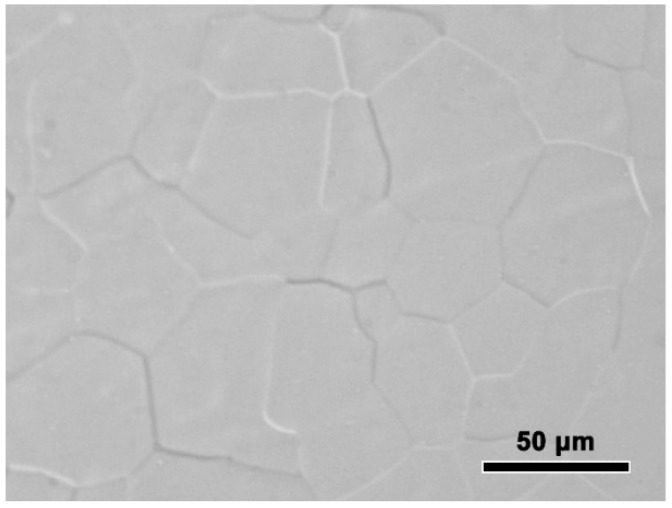
Optical microstructure the rheo-extruded Al-Sc-Zr-Fe alloy wire.

**Figure 3:**
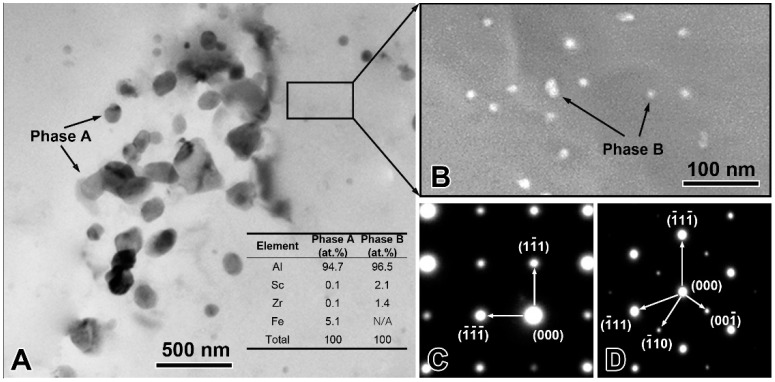
Transmission electron microscopy (TEM) microstructure and composition identification of the precipitates in the rheo-extruded Al-Sc-Zr-Fe alloy wire. TEM microstructure and EDS spectroscopy (**A**,**B**); selected area electron diffraction (SAED) pattern of the plate-shape phase A (**C**); and SAED pattern of the spherical phase B (**D**).

The experimentally measured composition of Al-Sc-0.1 Zr-0.2 Fe alloy was: Sc 0.050 ± 0.003 mass%, Zr 0.101 ± 0.003 mass%, Fe 0.205 ± 0.002 mass%, and balanced Al. The loss of alloying elements was 0.006 mass% Al by surface oxidation during melting process. The optical microstructure the rheo-extruded Al-Sc-Zr-Fe alloy wire is shown in Figure 2. The tensile strength, conductivity, and elongation of the rheo-extruded wire was 102.5 MPa, 58.70% IACS, and 28.45%, respectively. The TEM microstructure, energy dispersive spectroscopy (EDS) and selected area electron diffraction (SAED) patterns of the second phases in the alloy wire are presented in Figure 3. Two kinds of second phases with different morphologies were observed in the alloy wire. A plate-shape phase with an average length of 300 nm is shown in Figure 3A, and another fine spherical phase with the diameter of 15 nm disperses in the matrix is shown in Figure 3B (a local amplification in Figure 3A). The EDS and SAED patterns analysis were performed on the plate-shape phase and spherical phase in the alloy wire, respectively. A large number of Al and Fe elements as well as a small amount of Sc and Zr (Figure 3A) were detected in the plate-shape phase, and the SAED pattern was identified as the diffraction pattern of Al_3_Fe phase along the [−101] zone axis (Figure 3C). For the spherical phase, the EDS spectroscopy revealed that there are Al, Sc, and Zr elements in this phase, and Fe element was not detected in it (Figure 3A). The SAED pattern in Figure 3D was identified as the diffraction pattern of Al_3_(Sc, Zr) phase along the [−101] zone axis.

**Figure 4:**
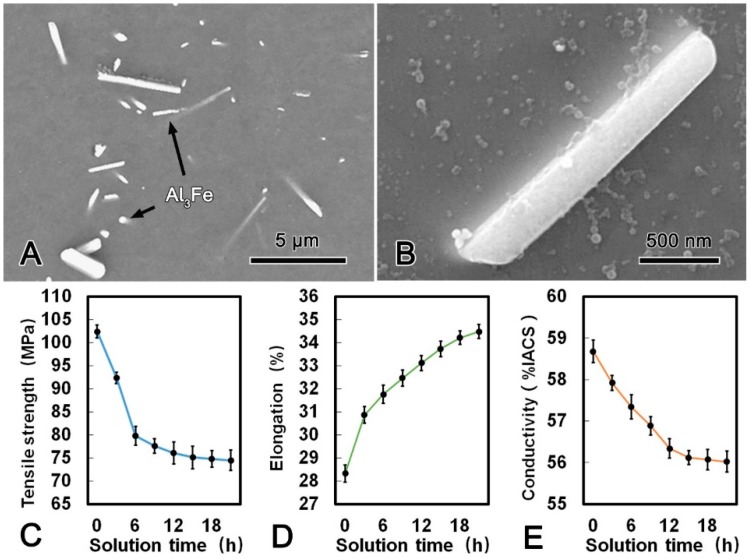
SEM microstructure of the alloy wire subjected to solid solution treatment (SST) (**A**); SEM image of a Al_3_Fe phase in SST processed alloy (**B**); tensile strength (**C**); elongation (**D**); and conductivity (**E**) of SST processed alloy.

Figure 4A shows the microstructure of the alloy wire treated at 630 °C for 21 h. There were some obvious changes of the morphology and distribution of the second phases after SST. Most of the Al_3_Fe phase with dimensions of 3 μm and 0.5 μm transformed into rod-shape in the longitudinal and transverse direction, respectively. Meanwhile, no Al_3_(Sc, Zr) phase was observed from the TEM micrograph, which indicated that all Al_3_(Sc, Zr) phase has dissolved into the matrix. When the SST time extended to 6 h and 21 h, the morphology of Al_3_Fe phase has transformed from plate-shape into rod-shape attributed to the diffusion of Fe atoms (Figure 4B). The mechanical and conductive properties of the alloy wire during SST are summarized in Figure 4C–E. Both the tensile strength and conductivity decreased firstly and then tended to be stable, while the elongation increased continuously with the prolonging of the SST time. The tensile strength, conductivity, and elongation of the alloy wire reached 75.1 MPa, 56.12% IACS, and 33.74%, respectively. The corresponding decrement in the tensile strength and conductivity were 26.7% and 4.4%, respectively. However, the elongation was 19.0% which higher than that of the CRE alloy wire.

**Figure 5:**
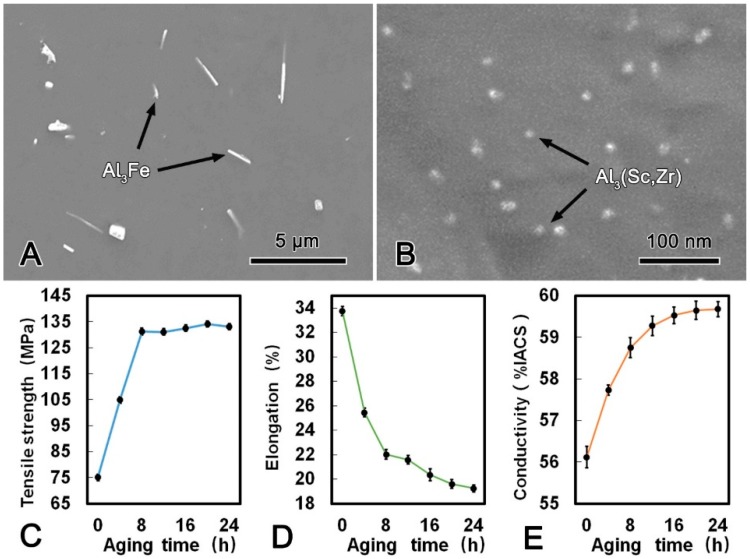
SEM microstructure of the alloy wire subjected to solution treatment followed by aging treatment (SAT) (**A**); SEM image of Al_3_(Sc, Zr) phases in SAT processed alloy (**B**); tensile strength (**C**), elongation (**D**); and conductivity (**E**) of SAT processed alloy.

The microstructure of the alloy wire subjected to SAT at 300 °C for 24 h are shown in Figure 5. It can be observed that the morphology and average length of the Al_3_Fe phase had no obvious change compared with those in the SST alloy, and the spherical Al_3_(Sc, Zr) phase with the diameter of 15 nm precipitates uniformly in the matrix. As shown in Figure 5C–E, the mechanical and conductive properties of the alloy wire during SAT are summarized. The tensile strength and conductivity increased rapidly during the first 3 h and then reached the stable status whilst the elongation decreased. The maximum conductivity was 59.68% IACS when the alloy wire subjected to SAT at 300 °C for 24 h, and the tensile strength and elongation was 134.1 MPa and 19.2%, respectively.

**Figure 6:**
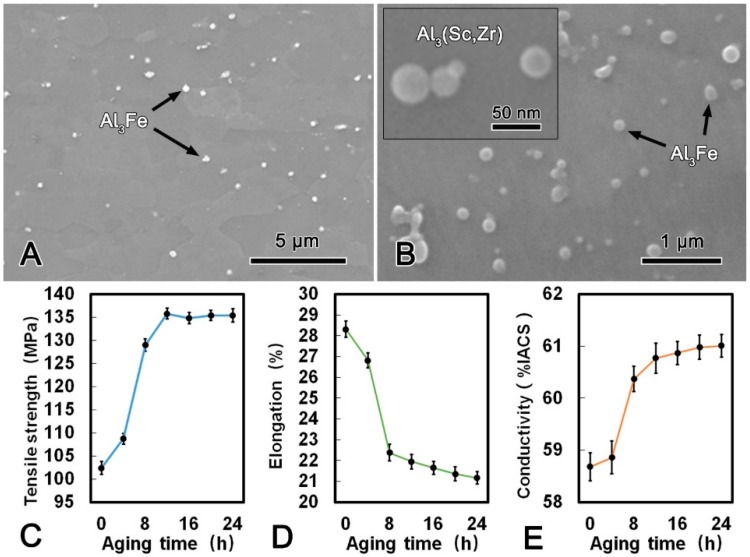
SEM microstructure of the alloy wire subjected to direct aging treatment (DAT) (**A**); SEM image of a Al_3_Fe and Al_3_(Sc, Zr) phases in DAT processed alloy (**B**); tensile strength (**C**), elongation (**D**); and conductivity (**E**) of DAT processed alloy.

The microstructure images of the alloy wire after DAT at 300 °C for 24 h and the mechanical and conductive performance are shown in Figure 6. Compared with the microstructure of the CRE alloy wire, it is notable that the Al_3_Fe phase in the DAT alloy wire was refined significantly and changed to spherical shape with a diameter of ca. 200 nm. Meanwhile, the size and morphology of the Al_3_(Sc, Zr) phase did not change obviously after the DAT. The metallographic structures of the alloy after treated at 300 °C for 24 h (Figure 6B) illustrates that the large plate-shape Al_3_Fe phase gradually decomposed into smaller spherical phase, while the small Al_3_Fe phase turned spheroidized after the DAT process. The tensile strength increased rapidly in the first 6 h, and then kept constant. Conversely, the elongation decreased gradually. The maximum tensile strength of the DAT alloy wire was 135.8 MPa, which was 33% higher than that of the CRE alloy wire. The elongation was 21.4% that was 22% lower than that of the CRE alloy wire. Meanwhile, with increasing aging time, the conductivity increased significantly in the first 6 h and then increased slightly. When DAT ran for 3 h, the conductivity of the alloy wire exceeded 60.00% IACS and reached 61.03% IACS, which was 3.6% higher than that of the CRE alloy wire. The refinement and spheroidization of the Al_3_Fe phase could effectively decrease the harmful effect, which was the main cause of the better mechanical performances of DAT processed alloy. Due to the balanced mechanical and conductive properties, the DAT treated alloys were then cold-drawn to further improve their strength.

**Figure 7:**
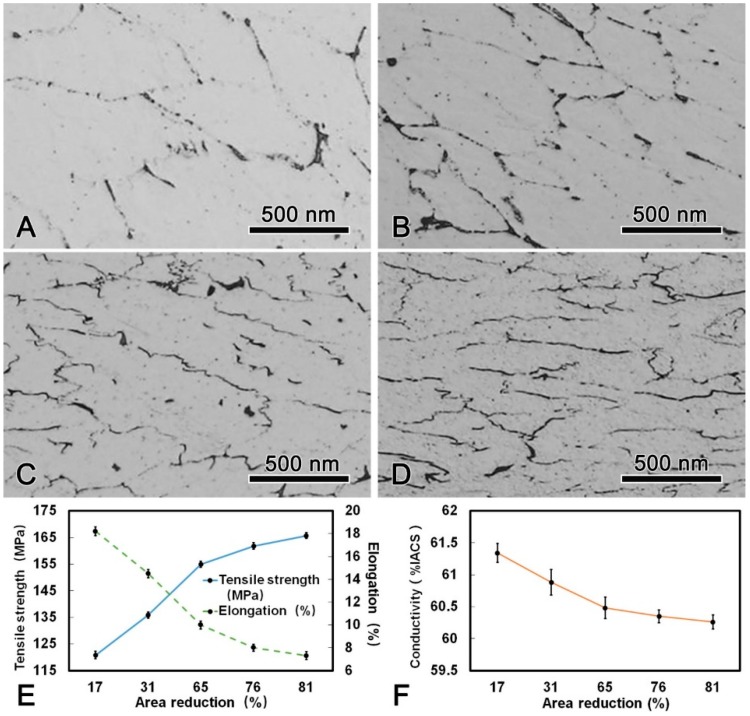
Microstructure of the DAT processed alloy wire after cold drawing with different area reduction: (**A**) 17%; (**B**) 31%; (**C**) 65%; (**D**) 81%; (**E**) changes of tensile strength, elongation, and (**F**) conductivity during cold drawing.

The microstructure, mechanical, and conductive properties of the DAT alloy wire after cold drawn with different area reduction were shown in Figure 7. It is obvious that the grains were elongated along the drawing direction and fibrous microstructures were formed after drawing process. The tensile strength increased with increasing area reduction, while the elongation decreased. The maximum tensile strength of the alloy wire after cold drawing was 165.7 MPa which was 22% higher than that of the DAT alloy wire. It is worth noting that with increasing drawing passes, the conductivity of the alloy wire decreased slightly, yet was still higher than 60.00% IACS during the whole process. When the DAT alloy wire was cold drawn to 4.5 mm in diameter, the tensile strength reached 165.7 MPa and the conductivity was 60.26% IACS. The thermal-resistant property test results showed that the residual strength were 95.42% and 94.56% when the alloy wire were held at 230 °C for 1 h and at 400 °C for 1 h, respectively. Allowable continuous operating temperature of the present alloy wire exceeded 230 °C, which was 80 °C higher than that of the AT1 type alloy wire according to the IEC international standard (162 MPa of tensile strength, 1.7% of elongation, and 60% IACS of conductivity) [16].

## 4. Discussion

During SST process at 630 °C, Fe atoms gradually diffused into the matrix, thus leading to the small Al_3_Fe phase dissolved and the morphology of large Al_3_Fe phase transformed into rod-shape (Figure 4). Based on Gibbs–Thomson theory [17], the concentration on the boundary of the small phase is greater due to the larger curvature. Therefore, the solute atoms would diffuse from the side of small phase to the side of large phase. As a result, the small Al_3_Fe phases in the alloy wire were dissolved, and the large Al_3_Fe phase grew up with solution time prolonging. Furthermore, due to the fine-plane morphology and the highly preferred orientation noted by Sha et al. [18], the Al_3_Fe phase tended to grow up along the close-packed (111) surface and formed a long rod-shape phase. The rod-shape Al_3_Fe phase had a harmful effect on the mechanical performance of the alloy wire. Meanwhile, during the solution process, static recovery eliminated the internal stress induced by CRE process, thus the alloy wire was softened. It is worth emphasizing that all the Al_3_(Sc, Zr) phase dissolved into the matrix after SST as shown in Figure 4, and the solution strengthening of Al_3_(Sc, Zr) phase was weaker than that of precipitation strengthening [19]. Therefore, the dissolution of Al_3_(Sc, Zr) phase had a softening effect on the alloy wire. The mechanical and conductive properties of the alloy wire begin to stabilize when the static recovery was accomplished (Table 3).

During the first 3 h of the SAT, the coherent spherical Al_3_(Sc, Zr) phase with an average size of 15 nm participated rapidly from the matrix. Due to dislocations and grain boundaries could be pinned effectively by the dispersed nano-sized Al_3_(Sc, Zr) phase, and tensile strength of the alloy wire was improved accordingly. Meanwhile, the Al_3_(Sc, Zr) phase inhibited the recrystallization of the alloy wire at high temperature and reduced the softening effect of the alloy wire in heat treatment process. The Al_3_Fe phase in the DAT alloy wire was obviously refined due to two main reasons. Firstly, the higher energy of the large Al_3_Fe phase with larger surface area had an effect on the diffusion of solute atoms around from thermo-dynamical point of view [10]. Thus, the coarse Al_3_Fe phase transformed into smaller spherical phase to improve stability when the dynamic conditions were satisfied. The disintegration occurred mainly at the concaves and gaps on the surface of Al_3_Fe phase, the segregation regions of Fe atoms. The concentrations of Fe atoms were higher in these regions and promoted the diffusion of Fe atoms to form necking [20]. Secondly, according to the principle of minimum surface energy, the small Al_3_Fe phase had a tendency to transform into spherical-shape phase with a minimum surface area, which drove Fe atoms diffuse from the sharp angles to flat areas [21]. In addition, Fe atoms in the angle point of Al_3_Fe phase were also in a high energy state, thus they showed a tendency to diffuse to other positions.

The refinement and spheroidization of the Al_3_Fe phase could effectively decrease its embrittlement effect on the alloy, which was the main cause of the better mechanical performances of DAT processed alloy (Table 3). The conductivity of the DAT alloy wire was also obviously increased compared with that of the CRE alloy wire because of the static recovery. Refinement of Al_3_Fe phase and further precipitation of Sc and Zr elements could reduce the crystal defects, and therefore reduced the scattering effect of the conductive electrons [22].

In the subsequent cold drawing deformation after DAT, the alloy wire was hardened due to the increase of dislocation density (Figure 7). For Al alloys, due to the high stacking fault energy and the narrow width of extended dislocation, the cross glide of dislocations leads to dislocations pile-up and even form dislocation walls [23]. Moreover, the movable dislocations would be effectively pinned by the dispersed Al_3_(Sc, Zr) phase when the alloy wire undergo deforming [5,19]. A greater external force was required for the dislocations to cross these obstacles, due to which the strength was increased while the elongation was decreased [24]. It is worth noting that the conductivity of the alloy wire along the drawing direction decreased slightly during cold drawing. This is because the equiaxed grains were elongated with the increase in the number of drawing passes, forming deformation bands in the drawing direction [22]. With the increase in the dislocation and vacancy densities, the electronic scattering effect was enhanced, resulting in a decreasing conductivity of the alloy wires [25].

## 5. Conclusions

Low-cost heat-resistant Al-Sc-Zr-Fe conductor wire was successfully manufactured by CRE process. The mechanical and conductive properties of the materials after different heat treatment and mechanical deformation methods were analysed and the following essential conclusions can be obtained:

(1) The plate-shape Al_3_Fe phase in the CRE Al-Sc-Zr-Fe alloy conductor transformed into rod-like and elongated during SST at 630 °C with a length of 300 nm. In comparison, the Al_3_Fe phase was significantly refined to 200 nm in diameter and spheroidized during DAT at 300 °C.
(2) Both the mechanical and conductive properties of the alloy were significantly improved after aging treatment due to uniformly distributed Al_3_Fe precipitate with average diameter of 15 nm, which reduced scattering effect of the conductive electrons. The following cold drawing process led to significant increase in tensile strength whilst slight decrease in conductivity and drop in elongationdue to formation of dislocation walls.
(3) Cold-drawing after DAT was proved the optimized process to abtain the favorable mechanical and conductive properties. The tensile strength, elongation, and conductivity of the alloy conductor wire after optimized thermos-mechanical treatment and cold drawing process reached 165.7 MPa, 7.3%, and 60.26% IACS, respectively. The thermal resistance of this conductor wire is superior to that of the standard AT1 type alloy conductor according to IEC international standard.

## Figures and Tables

**Table 1:** Heat treatment parameters.

	Time (h)						
Solid Solution at 630 °C (in SST and SAT)	0	3	6	9	12	18	21
Aging at 300 °C (in SAT and DAT)	0	4	8	12	16	20	24

**Table 2:** Area reduction of the wires after different cold drawing passes.

Diameter (mm)	Area Reduction (%)
10.5	0
9.5	17
8.5	31
6	65
5	76
4.5	81

**Table 3:** The optimal mechanical and conductive properties of the alloy after different process.

Process	CRE	SST	SAT	DAT	DAT + Cold Drawing
Tensile Strength (MPa)	102.5	75.1	134.1	135.8	165.7
Elongation	28.45%	33.74%	19.20%	21.40%	7.63%
Conductivity (% IACS)	58.70	56.12	59.68	61.03	60.26
